# A structured learning curve for visuomotor recalibration and surgical performance during transition from two-dimensional to three-dimensional laparoscopic radical cystectomy

**DOI:** 10.3389/fonc.2026.1811010

**Published:** 2026-09-16

**Authors:** Xiaodong Qing, Xiangzheng Wu, Wenbo Gao

**Affiliations:** 1 Department of Surgery, Ningbo Integrated Traditional Chinese and Western Medicine Hospital, Ningbo, Zhejiang, China; 2 Department of Urology, Ningbo Integrated Traditional Chinese and Western Medicine Hospital, Ningbo, Zhejiang, China

**Keywords:** bladder cancer, cystectomy, laparoscopy, learning curve, three-dimensional, visuomotor recalibration

## Abstract

**Background:**

Three-dimensional (3D) laparoscopy offers stereoscopic depth perception, but moving from conventional two-dimensional (2D) systems calls for a period of visuomotor adaptation. How this learning curve unfolds in complex oncological procedures like laparoscopic radical cystectomy (LRC) has not been well characterized.

**Methods:**

This single-surgeon retrospective cohort study included 140 consecutive patients who underwent LRC by an experienced surgeon (>300 prior 2D procedures). Cases were divided chronologically into one 2D group (the last 50 2D cases) and three consecutive 3D groups (3D-1 to 3D-3, 30 cases each). The primary endpoint was pelvic-phase operative time- the vision-dependent part of dissection. Secondary outcomes included estimated blood loss (EBL), lymph node yield, positive margin rate, major complications (Clavien-Dindo ≥II), and surgeon-rated stereoscopic comfort and hand-eye coordination (visual analogue scales).

**Results:**

Baseline characteristics were similar across groups. Change-point analysis identified a single significant transition point at case 31 (95% CI: 28-34). Pelvic-phase time in 3D-1 group (median 198 min) was comparable to the 2D reference (185 min, *P*> 0.05), marking an adaptation phase. After that, time fell sharply to 155 min in 3D-2 group and 152 min in 3D-3 group (both *P*< 0.001 *vs.* groups 2D and 3D-1). EBL dropped from 450 mL (3D-1) to 250 mL (3D-3) (*P* < 0.05). Lymph node yield, positive margin rates, and major complications remained stable. Subjective comfort and coordination scores improved steadily across the 3D groups (both *P* < 0.001) and correlated negatively with pelvic-phase time (*r_s_* = -0.68, *P* < 0.001).

**Conclusions:**

Transitioning from 2D to 3D LRC involves a distinct learning curve of about 30 cases. Once visuomotor recalibration is achieved, operative efficiency and intraoperative safety improve substantially without compromising pathological quality. These findings offer an evidence-based framework for structured training and technology adoption in complex pelvic oncology.

## 1 Introduction

The evolution of minimally invasive surgery has been closely tied to advancement in visualization technology (1). Conventional two-dimensional (2D) laparoscopy, despite its widespread adoption, suffers from an inherent limitation: the lack of binocular depth cues. In the absence of binocular depth cues, surgeons must compensate by using monocular visual cues (such as motion parallax, shadow, and relative size) alongside cognitive inference to assess spatial relationships. This compensatory process heightens cognitive load and can undermine surgical precision, especially in complex procedures (2). The introduction of three-dimensional (3D) laparoscopy represents a significant technological advance, as it restores stereoscopic vision and provides intuitive depth perception (3). These benefits are especially valuable in complex pelvic oncological surgeries, such as laparoscopic radical prostatectomy (LRP) and laparoscopic radical cystectomy (LRC), where delicate dissection within a confined anatomical space is critical for both oncological radicality and functional preservation.

Despite the clear theoretical advantages of 3D systems, the transition from 2D to 3D laparoscopy is not seamless. Conventional 2D imaging forces surgeons to compensate for the absence of stereopsis by relying on monocular cues and learned compensatory strategies, which therefore impose high cognitive load (4). The restoration of binocular depth cues when transitioning from 2D to 3D technology, while beneficial, requires a period of visuomotor recalibration- during which the surgeon’s perceptual and motor systems adapt to the new visual depth information and integrate it into smooth, efficient surgical maneuvers. This adaptation is not instantaneous but occurs gradually over a series of procedures (5). However, existing comparative studies on 2D versus 3D laparoscopy have predominantly used cross-sectional designs or global operative metrics that might fail to isolate the vision-dependent components of operations (6, 7). Consequently, the longitudinal adaptation process in experienced surgeons transitioning between visualization systems remains poorly characterized, and the relationship between subjective visuomotor perception and objective surgical performance has not been rigorously defined.

Therefore, in this study, we aimed to characterize the learning curve associated with the transition from 2D to 3D LRC using a structured, phase-specific approach. By isolating the visual-dependent pelvic dissection phase and integrating both objective surgical outcomes and subjective visuomotor metrics, we sought to characterize the adaptation process and elucidate the mechanisms of performance improvement.

## 2 Materials and methods

### 2.1 Study design and patient population

This was a single-surgeon, retrospective cohort study designed to evaluate the learning curve associated with the transition from 2D to 3D laparoscopy in radical cystectomy. The study was conducted at a tertiary hospital between January 2020 and September 2025. All procedures were performed by one senior surgeon (W.G.) who had completed over 300 independent 2D-LRC operations before adopting 3D technology. The study was reported in accordance with the STROBE guidelines, and all patients gave written informed consent. The study was approved by the Institutional Ethics Committee of our hospital in compliance with the Declaration of Helsinki, and written informed consent was obtained from all subjects before their participation.

A total of 165 consecutive patients undergoing LRC were initially screened. Inclusion criteria were: (1) histologically confirmed bladder urothelial carcinoma without distant metastasis; (2) planned LRC with standard pelvic lymph node dissection; (3) complete perioperative records and pathological reports. Exclusion criteria were: (1) conversion to open surgery; (2) incomplete records; and (3) follow-up <30 days. Eventually, 25 patients were excluded (10 conversions, 8 missing data, 7 insufficient follow-up), and 140 cases were enrolled for final analysis (**Table 1**).

**Table 1:** Patient screening and reasons for exclusion (*n* = 165).

Reason category	Specific reason	*n*	Percentage (%)
Intraoperative event	Conversion to open surgery*	10	6.1
Missing data	Incomplete perioperative records	8	4.8
Follow-up insufficient	Postoperative follow-up <30 days	7	4.2
Total excluded		25	15.2

Final analytic cohort: 140 patients.

*Distributed chronologically: 6 during the 3D-1 period (cases 1–30), 3 during the 3D-2 period (cases 31–60), and 1 during the 3D-3 period (cases 61–90).

The enrolled patients were divided chronologically into four groups based on the surgical sequence and visualization mode: 2D group (*n* = 50): the final 50 consecutive 2D-LRC procedures performed before the first 3D case; 3D-1 group (*n* = 30): the first 30 consecutive 3D-LRC procedures (cases 1-30); 3D-2 group (*n* = 30): the next 30 3D-LRC procedures (cases 31-60); 3D-3 group (*n* = 30): the subsequent 30 3D-LRC procedures (cases 61-90). All operations followed a standardized radical cystectomy protocol. Moreover, urinary diversions were performed extracorporeally; and the urinary diversion type (ileal conduit or cutaneous ureterostomy) was chosen according to clinical indication and patient condition. Orthotopic neobladder was not performed due to institutional technical limitations. The flowchart of atient enrollment and group assignment is shown in **Figure 1**.

**Figure 1:**
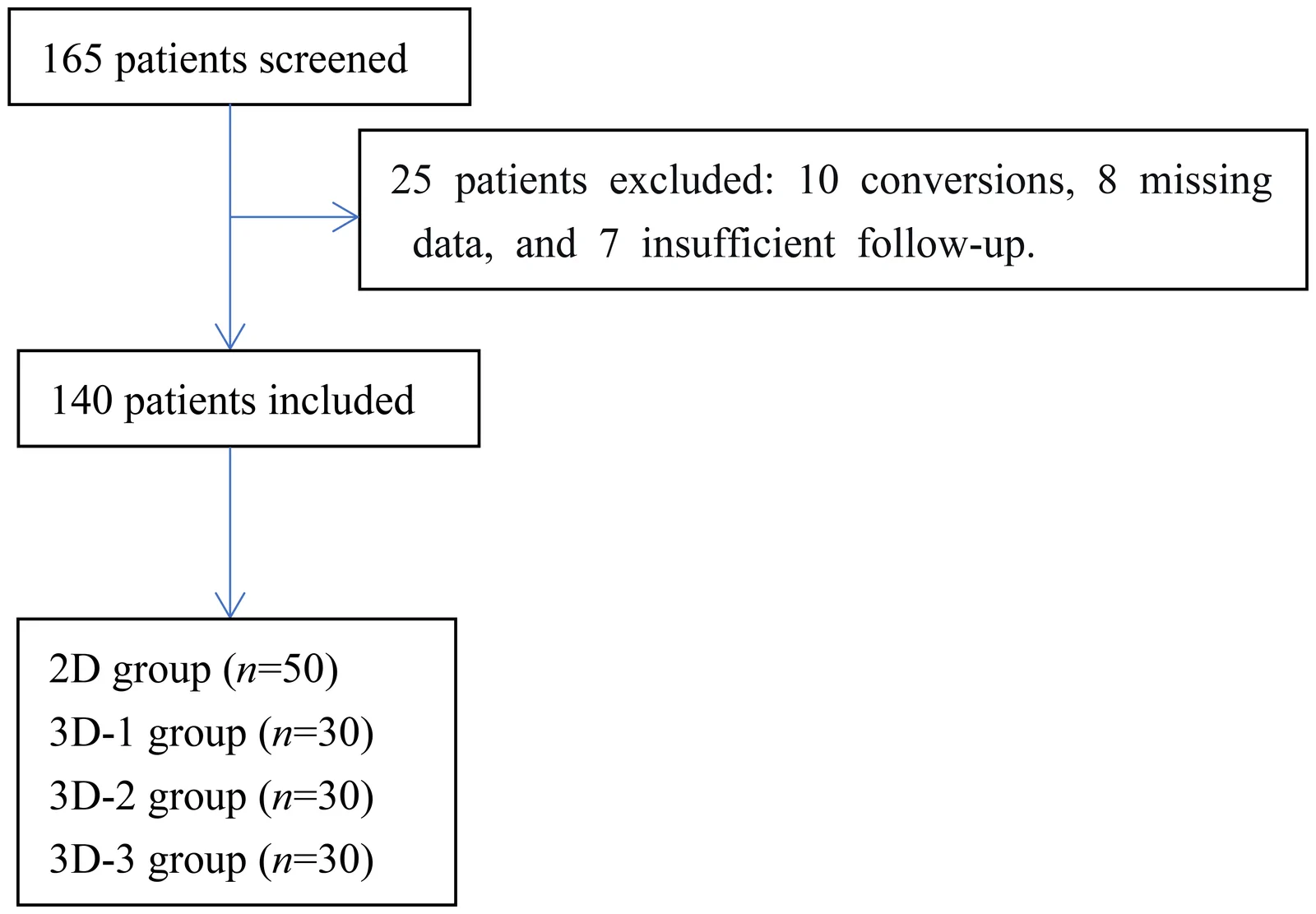
Patient enrollment and group assignment.

### 2.2 Outcome measures

The primary endpoint was pelvic-phase operative time, defined as the interval from pneumoperitoneum establishment to complete resection of the bladder/pelvic organs plus standard pelvic lymph node dissection. This phase was selected because it is highly dependent on depth perception and hand-eye coordination under laparoscopic vision. Secondary endpoints included estimated blood loss (EBL), total lymph node yield, positive soft tissue margin rate, complications (Clavien-Dindo grade ≥II) within 30 days, postoperative hospital length of stay (LOS), and subjective assessments (stereoscopic comfort and hand-eye coordination scores). Surgeon-rated VAS scores for stereoscopic comfort and hand-eye coordination were recorded by the surgeon immediately after each operative procedure (within 15 minutes of skin closure), before reviewing the patient’s outcomes. All surgical procedures followed a standardized five-port transperitoneal approach, with a schematic diagram shown in **Figure 2**. The 3D laparoscopic system applied was the Olympus 3D L.O.S. platform (Olympus Medical Systems, Tokyo, Japan), with standard 5-mm and 10-mm laparoscopic instruments. The surgical team (assistant and scrub nurse) remained consistent throughout the study period. The surgeon had no prior 3D laparoscopic experience before this series.

**Figure 2:**
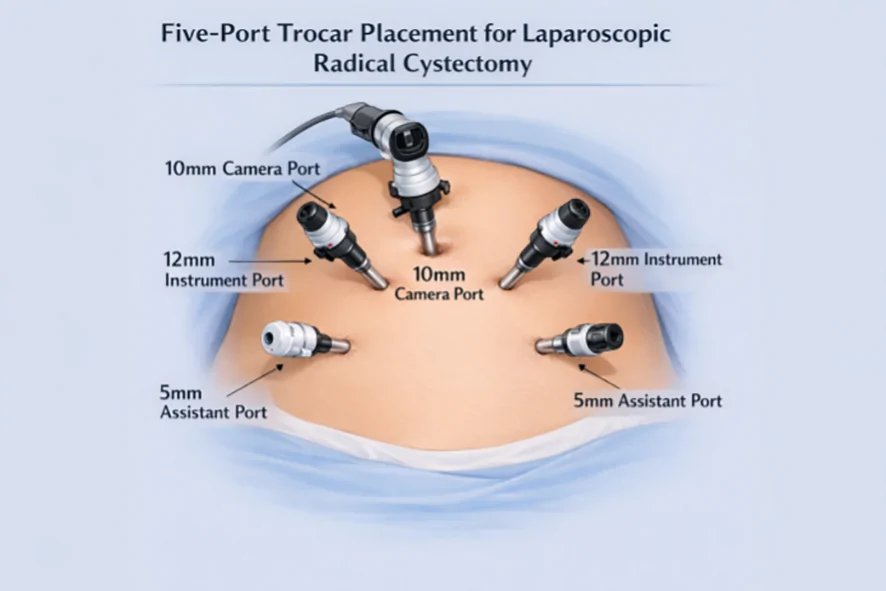
Schematic diagram of the five-port transperitoneal approach used for laparoscopic radical cystectomy.

### 2.3 Statistical analysis

Continuous variables were tested for normality using the Shapiro-Wilk test. Normally distributed data are presented as mean ± standard deviation (SD), non-normally as median and interquartile range (IQR). Categorical variables are shown as counts and percentages.

Group comparisons (2D, 3D-1, 3D-2 and 3D-3) were performed using one-way analysis of variance (ANOVA) or Kruskal-Wallis test for continuous variables, and chi-square test for categorical variables, with Jonckheere-Terpstra test for trend. Spearman’s correlation assessed the relationship between subjective coordination scores and pelvic-phase operative time. All tests were two-sided, with *P* < 0.05 being considered statistically significant. Analyses were conducted using SPSS version 22.0 (SPSS Inc., Chicago, IL, USA). To objectively determine the proficiency threshold, change-point analysis was performed using the PELT (Pruned Exact Linear Time) algorithm on the continuous sequence of pelvic-phase operative times for the 90 consecutive 3D cases, implemented in R version 4.2.0 (changepoint package).

## 3 Results

### 3.1 Study flow and baseline comparability

Among 165 consecutive LRC procedures, 140 met the inclusion criteria. The 10 conversions were distributed chronologically as follows: 6 occurred during the period corresponding to the 3D-1 series (cases 1-30), 3 during the 3D-2 period (cases 31-60), and 1 during the 3D-3 period (cases 61-90). No conversions occurred in the 2D reference series. The four chronologically defined groups (2D, 3D-1, 3D-2, 3D-3) showed no significant differences in age, sex, BMI, American Society of Anesthesiologists (ASA) score, clinical T stage, or type of urinary diversion (all *P*>0.05), demonstrating intergroup comparability regarding baseline characteristics (**Table 2**).

**Table 2:** Baseline and pathological characteristics of the study groups (mean ± SD or *n* [%]).

Characteristic	2D (*n* = 50)	3D-1 (*n* = 30)	3D-2 (*n* = 30)	3D-3 (*n* = 30)	P (global)	*P* for trend
Age (years)	68.2 ± 7.5	67.5 ± 8.1	68.9 ± 6.9	66.8 ± 7.2	0.451	0.312
Male, *n* (%)	42 (84.0)	25 (83.3)	26 (86.7)	27 (90.0)	0.892	0.398
BMI (kg/m²)	24.7 ± 2.4	25.2 ± 2.8	24.2 ± 2.5	24.9 ± 2.3	0.387	0.564
Clinical T stage (≥T3), *n* (%)	12 (24.0)	6 (20.0)	7 (23.3)	5 (16.7)	0.845	0.451
Clinical N+ stage, *n* (%)	6 (12.0)	4 (13.3)	5 (16.7)	3 (10.0)	0.876	0.523
Pathological T stage (≥T3), *n* (%)	14 (28.0)	8 (26.7)	9 (30.0)	7 (23.3)	0.934	0.512
Pathological N+ stage, *n* (%)	8 (16.0)	5 (16.7)	6 (20.0)	4 (13.3)	0.891	0.482
ASA ≥III, *n* (%)	18 (36.0)	12 (40.0)	9 (30.0)	10 (33.3)	0.883	0.512
Ileal conduit, *n* (%)	43 (86.0)	25 (83.3)	26 (86.7)	27 (90.0)	0.891	0.482
Prior abdominal surgery, *n* (%)*	15 (30.0)	9 (30.0)	8 (26.7)	7 (23.3)	0.912	0.425

*Supplementary variable for illustration; groups were comparable across all baseline features.

### 3.2 Learning curve for pelvic dissection

Change-point analysis of the continuous 90-case 3D sequence identified a single significant change point at case 31 (95% CI: 28- 34). Pelvic-phase operative time exhibited a biphasic learning pattern consistent with this threshold (**Figure 3**). During the first 30 3D cases (3D-1 group), median time was 198 min (IQR 175-225), statistically similar to the 2D group (185 min, IQR 170-205; *P* > 0.05), indicating an adaptation phase. Thereafter, time dropped sharply to 155 min (IQR 145-170) in 3D-2 group and plateaued at 152 min (IQR 140-165) in 3D-3 group (both *P* < 0.001 *vs.* 2D and 3D-1). No further reduction occurred between groups 3D-2 and 3D-3 (*P* = 0.743). In contrast, urinary diversion time remained unchanged across all groups (median 95-102 min, *P* = 0.321; **Figure 4**), confirming that the efficiency gain was specific to the laparoscopic visual-dependent phase.

**Figure 3:**
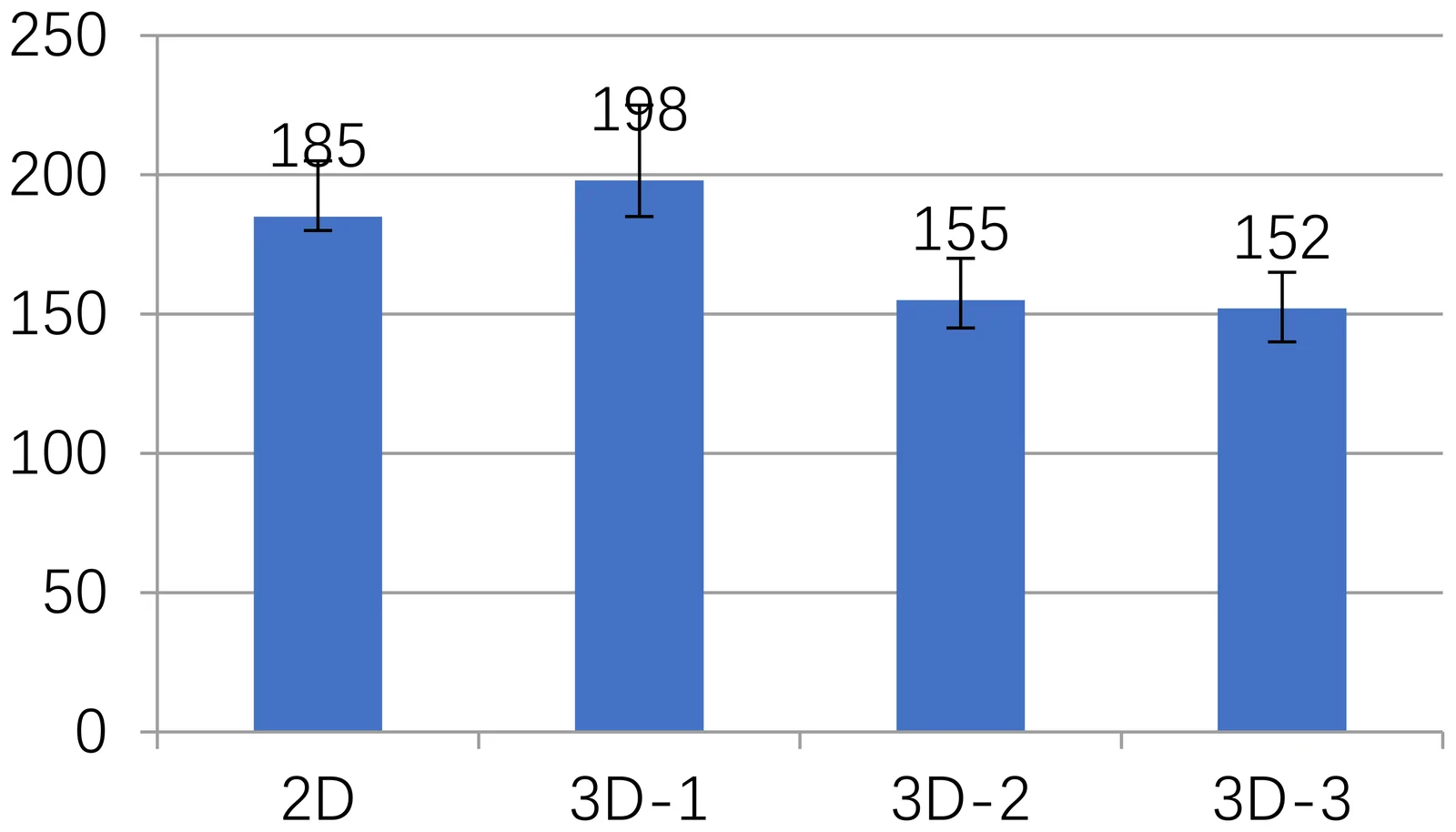
Pelvic-phase time (median, IQR). A significant reduction occurred after the initial 30-case adaptation period. *P* < 0.001 for groups 3D-2 and 3D-3 *vs.* groups 2D and 3D-1.

**Figure 4:**
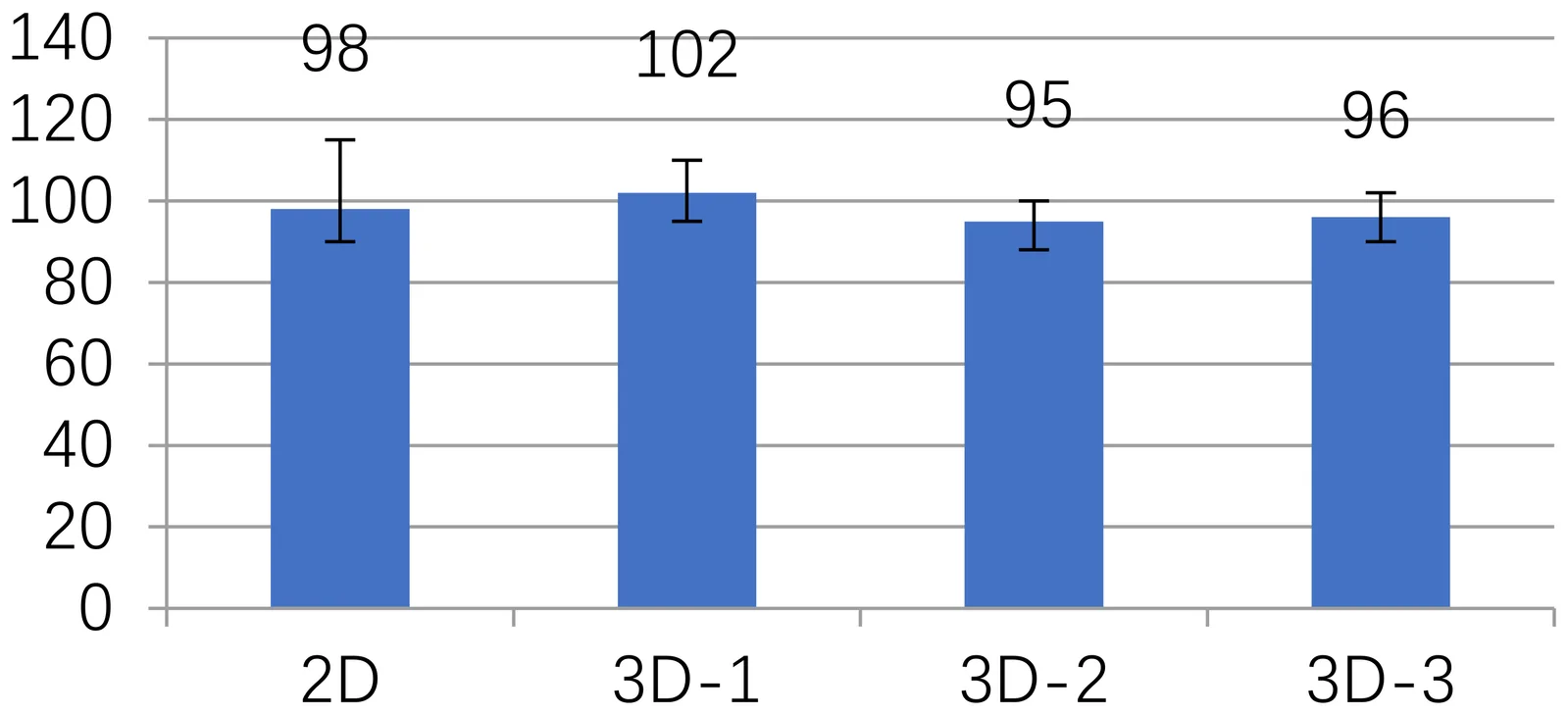
Urinary diversion time (median, IQR). No significant differences were observed across the groups 2D, 3D-1, 3D-2, and 3D-3 (*P* = 0.321).

### 3.3 Objective surgical and oncologic outcomes

EBL decreased progressively after the adaptation phase (**Figure 5**). Median EBL in 3D-2 group (300 mL, IQR 200- 400) and 3D-3 group (250 mL, IQR 200- 350) was significantly lower than in 2D (400 mL) and 3D-1 (450 mL) (pairwise *P* < 0.05). Meanwhile, lymph node yield remained stable across groups (median 18–19 nodes, *P* = 0.650; **Figure 6**), and positive surgical margin rates were low (0-6.7%, *P* = 0.432), suggesting preserved pathological radicality. Complication rates (Clavien-Dindo ≥II) were similar among groups (10.0- 23.3%, *P* = 0.541). Postoperative LOS showed a declining trend (from 12 days in 3D-1 group to 9 days in 3D-3 group) that did not reach statistical significance (*P* = 0.089; **Figure 7**). No 30-day mortality occurred. Detailed operative and postoperative outcomes are summarized in **Table 3**.

**Figure 5:**
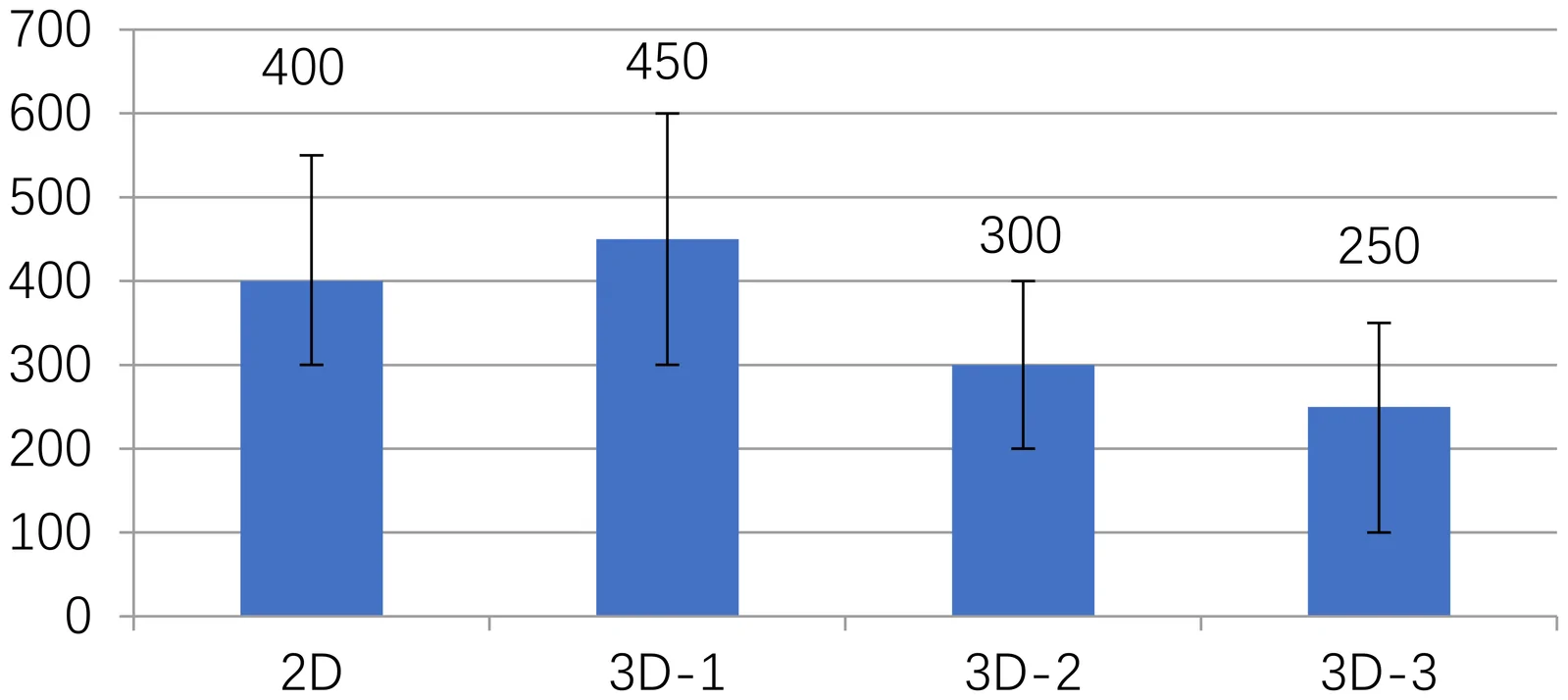
Estimated blood loss (median, IQR). A significant reduction occurred after the first 30 3D cases. *P* < 0.05 for groups 3D-2 and 3D-3 *vs.* groups 2D and 3D-1.

**Figure 6:**
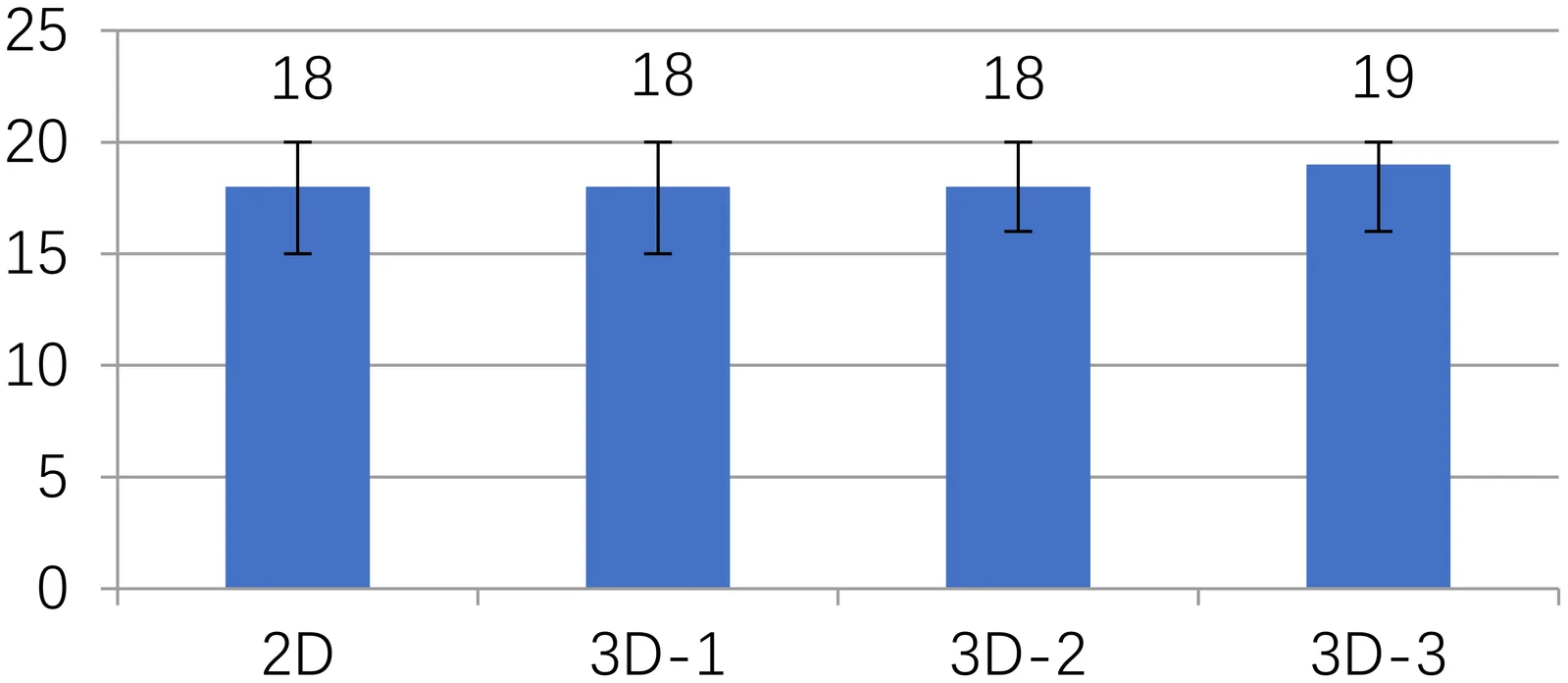
Lymph node yield (median, IQR). No significant differences were observed across the four groups (*P* = 0.650).

**Figure 7:**
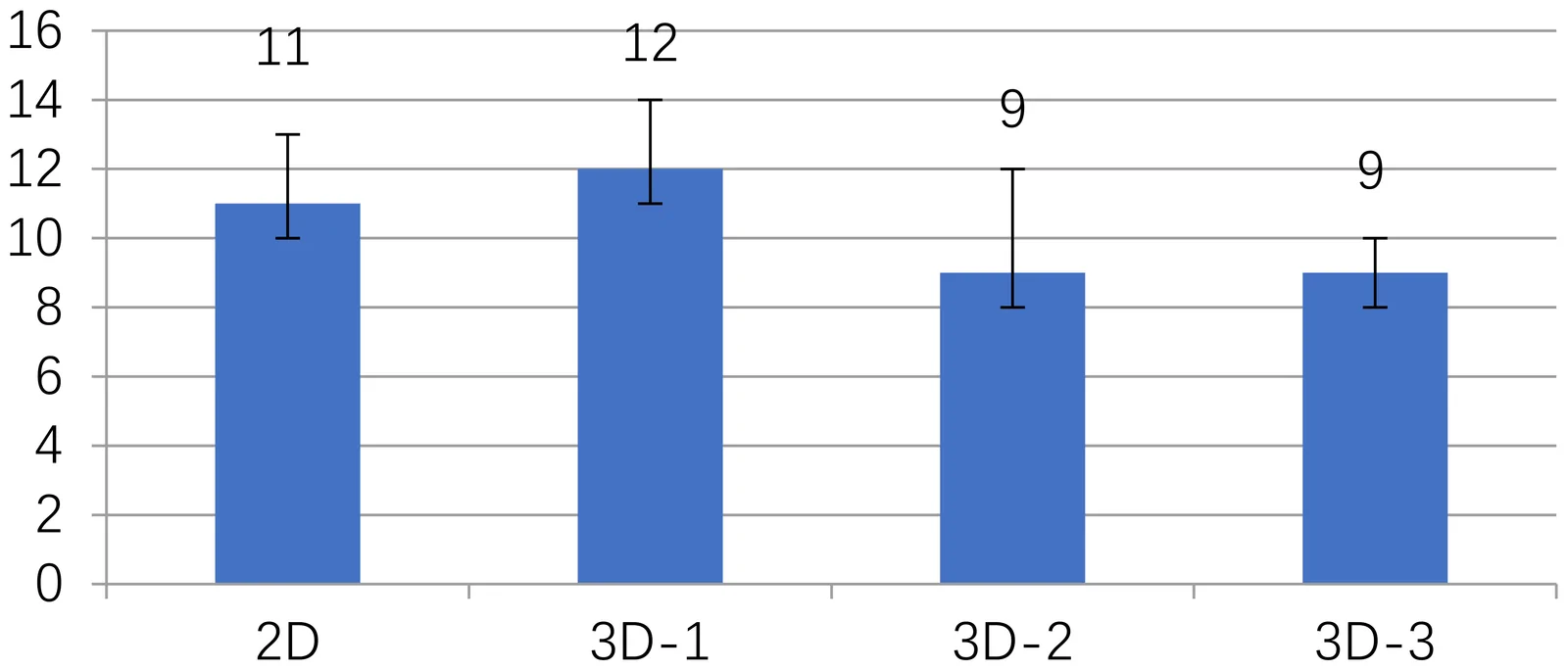
Postoperative hospital length of stay (mean ± SD). A decreasing pattern was observed from group 3D-1 to group 3D-3, although the difference did not reach statistical significance (*P* = 0.089).

**Table 3:** Operative and postoperative outcomes (median [IQR] or *n* [%]).

Variable	2D (*n* = 50)	3D-1 (*n* = 30)	3D-2 (*n* = 30)	3D-3 (*n* = 30)	*P* value
Pelvic-phase time (min)	185 [170-205]	198 [175-225]	155 [145-170]	152 [140-165]	<0.001
Urinary diversion time (min)	98 [90-105]	102 [95-110]	95 [88-100]	96 [90-102]	0.321
Total operative time (min)	283 [260-310]	300 [270-335]	250 [233-270]	248 [230-267]	<0.001
Estimated blood loss (mL)	400 [300-550]	450 [300-600]	300 [200-400]	250 [200-350]	<0.05
Transfusion rate, *n* (%)	12 (24.0)	9 (30.0)	5 (16.7)	4 (13.3)	0.267
Lymph node yield	18 [15-20]	18 [15-20]	18 [16-20]	19 [16-20]	0.650
Positive surgical margin, *n* (%)	2 (4.0)	2 (6.7)	1 (3.3)	0 (0)	0.432
Complications (Clavien-Dindo ≥II), *n* (%)	9 (18.0)	7 (23.3)	4 (13.3)	3 (10.0)	0.541
Complications (Clavien-Dindo ≥III), *n* (%)	3 (6.0)	2 (6.7)	1 (3.3)	1 (3.3)	0.812
Postoperative LOS (days), mean ± SD	11.3 ± 2.1	12.4 ± 2.5	9.2 ± 1.8	9.1 ± 1.6	0.089

### 3.4 Subjective visuomotor adaptation

Surgeon-rated stereoscopic comfort and hand-eye coordination scores improved markedly from 3D-1 group to 3D-3 group (**Figures 8**, **9**). Median comfort score rose from 4.5 to 8.0, and coordination score from 5.0 to 8.5 (both *P* < 0.001). A strong negative correlation was observed between coordination score and pelvic-phase time (*r_s_* = -0.68, *P* < 0.001), demonstrating a strong correlation between subjective visuomotor recalibration and objective surgical efficiency (**Table 4**).

**Figure 8:**
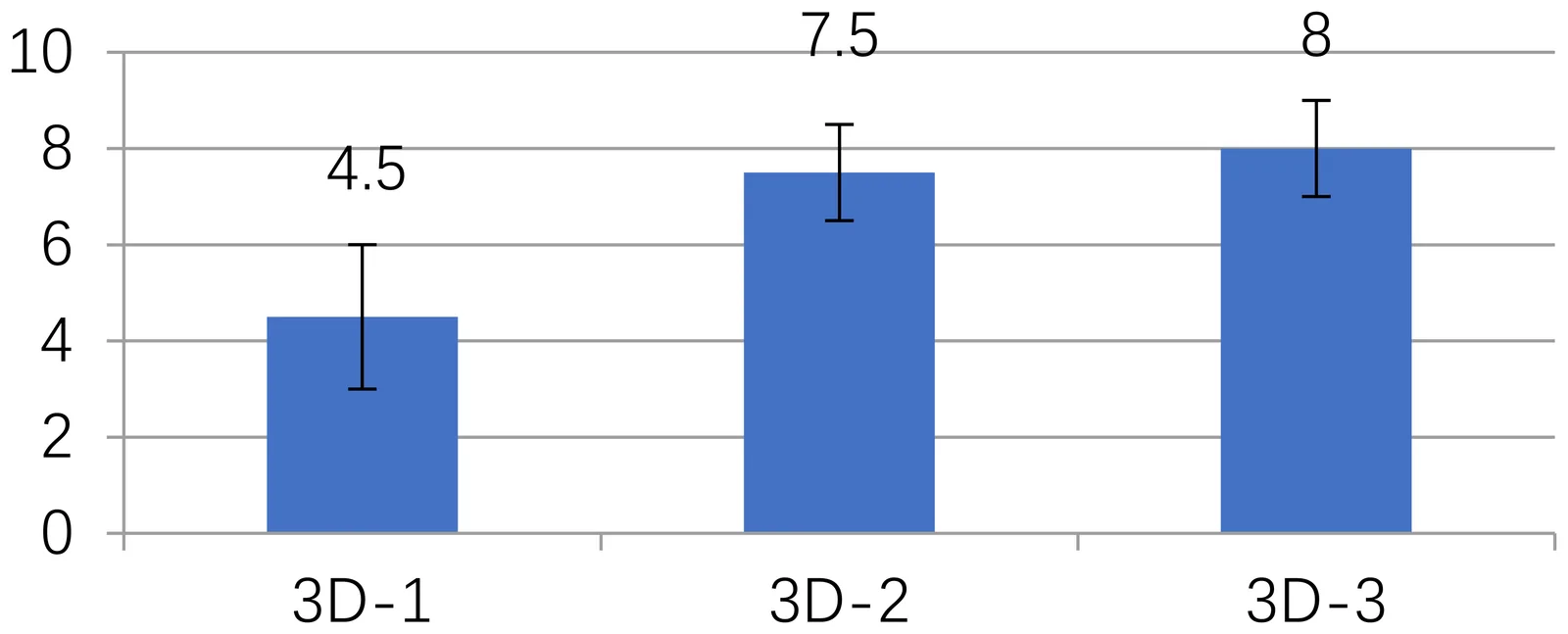
Surgeon-rated stereoscopic comfort scores (VAS, median with IQR). Scores improved significantly across the three 3D groups (*P* < 0.001).

**Figure 9:**
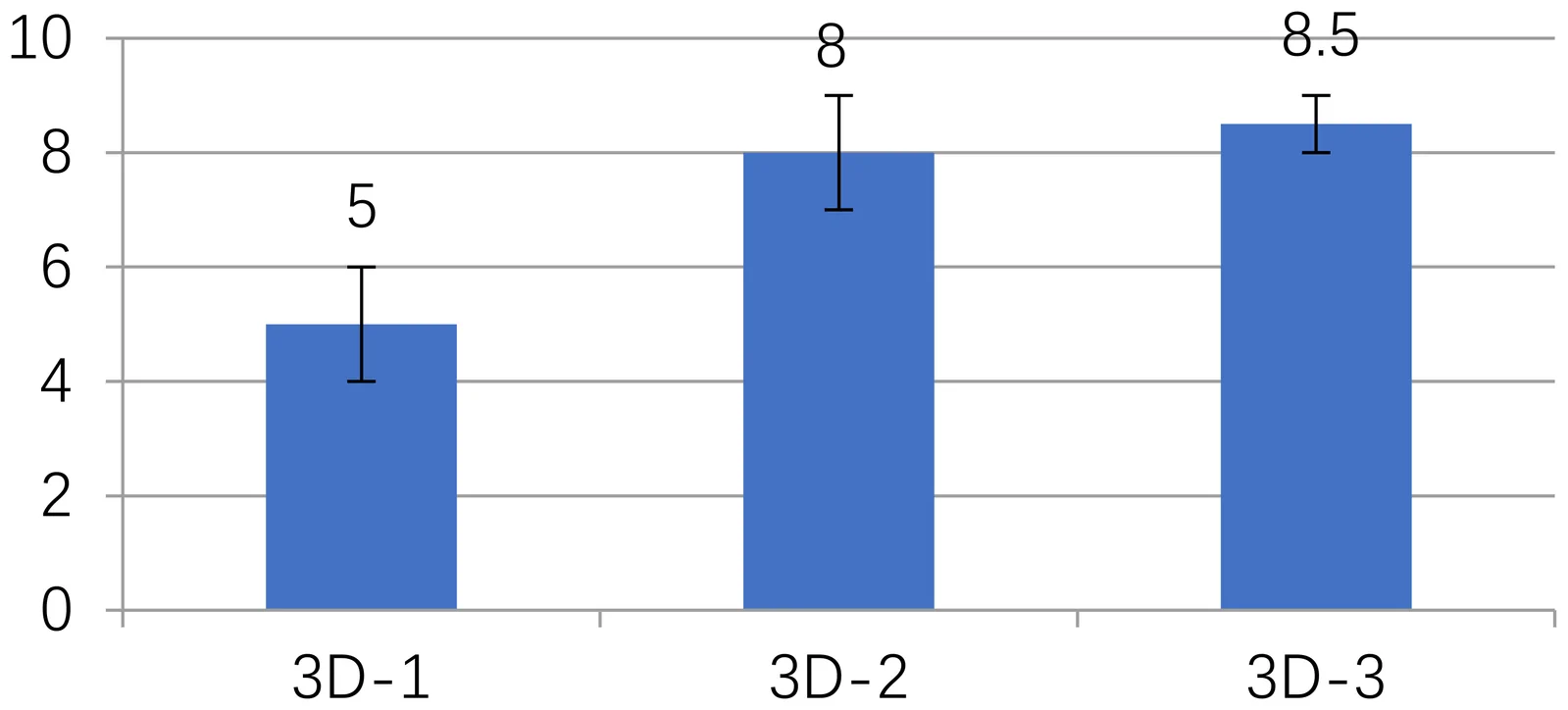
Surgeon-rated hand-eye coordination scores (VAS, median with IQR). Scores improved significantly across the three 3D groups (*P* < 0.001).

**Table 4:** Surgeon visual analogue scale (VAS) scores and correlation with pelvic-phase time.

Group	Stereoscopic comfort [median (IQR)]	Hand-eye coordination [median (IQR)]	Correlation coefficient (*r_s_*)*	*P* value
3D-1	4.5 [3.0-6.0]	5.0 [4.0-6.0]	–	–
3D-2	7.5 [6.5-8.5]	8.0 [7.0-9.0]	–	–
3D-3	8.0 [7.0-9.0]	8.5 [8.0-9.0]	–	–
Overall (all 3D cases)	–	–	-0.68	<0.001

*Spearman’s rank correlation between hand-eye coordination score and pelvic-phase operative time.

## 4 Discussion

The present study provides a structured, quantitative characterization of the learning curve associated with the transition from 2D to 3D LRC. Our key finding is that an experienced 2D surgeon requires approximately 30 cases to achieve visuomotor recalibration, after which operative efficiency (pelvic-phase time) and intraoperative safety (EBL) improve significantly, without compromising pathological quality. This learning curve follows a biphasic pattern: an initial adaptation phase (first 30 3D cases) with comparable performance to 2D, followed by a proficiency phase with sustained gains.

Our findings are consistent with and extending previous reports on 3D laparoscopy learning curves. In a simulator-based study using a pelvic trainer, Ozsoy et al. demonstrated that novice surgeons performed better with 3D than with 2D laparoscopy across multiple basic tasks, with significantly lower subjective workload and improved depth perception (8). Kang et al. observed a secondary learning curve when surgical novices transitioned between visualization systems, with proficiency achieved after approximately 26–30 repetitions of complex tasks (9). In clinical urology, Haapiainen et al. reported that the learning curve for urethrovesical anastomosis during 3D laparoscopic prostatectomy plateaued after around also 30 cases (4). In the present study, our results offered a novel, phased-time quantification of this transition specifically for LRC, isolating the visual-dependent dissection phase from the extracorporeal diversion step- an approach that had not been used in prior reports, to our knowledge. This design allows us to attribute the observed efficiency gains primarily to visuomotor recalibration rather than generalized surgical maturation. **Table 5** summarizes the comparison with prior studies.

**Table 5:** Comparison with previous 3D laparoscopy learning curve studies.

Study (year)	Procedure / model	Sample size (3D group)	Learning threshold (cases)	Primary endpoint	Phased-time analysis used?
Ozsoy et al. (2015) (8)	Pelvic trainer (novices)	25	No clear threshold	Task performance (basic tasks)	No
Kang et al. (2021) (9)	Laparoscopic task training	26	≈30	Task completion time	No
Haapiainen et al. (2021) (4)	3D laparoscopic prostatectomy	200	≈30	Urethrovesical anastomosis time	No
Present study	3D laparoscopic radical cystectomy	90	≈30	Pelvic-phase time, EBL, subjective coordination	Yes

To the best of our knowledge, no prior study has quantified this transition specifically for LRC with a phased-time analysis isolating the vision-dependent dissection phase from urinary diversion. Importantly, the current study was conducted by a surgeon with substantial prior 2D experience (>300 LRC procedures). This distinguishes our learning curve from *de novo* skill acquisition in novices and disentangles the pure visuomotor recalibration effect during technology transition from general surgical maturation. Moreover, consistent with our findings, a recent learning curve analysis of thulium laser en bloc resection of bladder tumors (TL-ERBT) also reported that a surgeon with extensive conventional transurethral resection of bladder tumor (TURBT) experience required approximately 32 cases to achieve proficiency, with significant reductions in operation time and conversion rates thereafter (10). This parallel observation across different surgical platforms supports the notion that visuomotor recalibration for novel endoscopic techniques follows a reproducible learning curve of around 30 cases when the operator has experienced baseline skills.

The concept of visuomotor recalibration offers a plausible framework for understanding our results. This process, defined as the brain’s ability to update the mapping between visual input and motor output in response to altered sensory feedback (11), is consistent with the adaptation observed in our study. The necessity of this recalibration is evident in the distinct learning curve we identified. While our design does not directly measure neurophysiological changes, the observed patterns- an initial plateau followed by a sharp improvement, specificity to the vision-dependent phase, and the correlation with subjective comfort, support the broader principle that stable visuomotor maps require substantial repetitive exposure (12, 13). Under 2D laparoscopic conditions, the surgeon lacks binocular depth cues, therefore must rely on monocular cues and cognitive inference to estimate spatial depth and relationships. The introduction of 3D vision restores stereopsis, allowing the brain to process depth information more naturally and automatically (14, 15). However, the transition is not instantaneous: as the surgeon’s visuomotor system must recalibrate to the new depth cues, a process that typically requires repetitive exposure (16). Our finding of a 30-case adaptation phase is in line with previous literature on perceptual learning, which suggested that 20–40 repetitions were often needed to achieve fluent, effortless performance in complex pelvic surgical procedures (17).

The strong negative correlation between hand-eye coordination scores and pelvic-phase time (*r_s_* = -0.68, *P* < 0.001) provides associative evidence linking subjective recalibration to objective efficiency. As the surgeon became more comfortable with stereoscopic depth, dissection became faster and more fluid. Notably, this improvement was specific to the laparoscopic phase. Urinary diversion time, which was performed extracorporeally under direct vision, remained unchanged. This dissociation reinforces that the learning curve is driven by visual adaptation, not by a general reduction in operative time across all steps. In addition, it is noteworthy that this adaptation occurred in a surgeon already proficient in 2D laparoscopy, underscoring that even experienced operators require dedicated recalibration when depth cues are restored.

The reduction in EBL after the adaptation phase (from a median of 450 mL in 3D-1 group to 250 mL in 3D-3 group) is clinically meaningful. In LRC, bleeding often occurs during dissection of the lateral pedicles and the dorsal vascular complex, where precise identification of tissue planes is critical (18, 19). Enhanced depth perception with 3D likely allows more accurate control of vascular structures, reducing inadvertent tears and the need for blind clamping. Importantly, this safety gain was not achieved at the expense of radicality. As shown in our study, lymph node yield remained stable (median 18–19 nodes) and positive margin rates were low (0-6.7%) across all groups. These pathological indicators suggested adequate radicality, indicating that 3D adoption does not appear to compromise the completeness of tumor resection.

Robotic-assisted radical cystectomy (RARC) offers superior three-dimensional visualization and wristed instrumentation (20), but its widespread adoption faces multiple barriers. These include high costs, infrastructure requirements, and regulatory constraints (e.g., China’s 2023 Catalog of Large Medical Equipment, which prioritizes robotic systems for selected high-tier hospitals (21, 22). In contrast, 3D laparoscopic systems are more affordable, portable, and require minimal additional training beyond the 30-case adaptation, as shown in our clinical study. For many institutions, transitioning from 2D to 3D laparoscopy may represent a cost-effective intermediate step that narrows the gap between conventional laparoscopy and robotics (23).

The recommendation that surgeons be supervised or proctored during the first 30 3D cases, while supported by our findings, should be considered hypothesis-generating. A single-surgeon retrospective study cannot establish a definitive training standard. Nonetheless, our data suggested that structured proctoring during this initial adaptation period might be beneficial to mitigate the risks associated with longer operative times.

### 4.1 Limitations

Several limitations warrant acknowledgment. First, this is a single-surgeon, retrospective study, which may limit its generalizability. The learning curve could differ for surgeons with less 2D experience in laparoscopic surgeries or for those in different healthcare settings. Second, the lack of randomization introduces potential selection bias, although baseline characteristics were well balanced in the current study. Third, the chronological design makes it difficult to definitively attribute all observed improvements solely to the 2D→3D transition; unmeasured time-varying factors (e.g., subtle improvements in team dynamics) cannot be entirely excluded. However, we confirmed that no formal changes were made to ERAS protocols, surgical equipment, or the core surgical team during the study period. Furthermore, the stability of the extracorporeal diversion time argues against a general temporal learning effect. Fourth, the sample size (*n* = 140) and relatively short follow-up period (30 days) are insufficient to detect differences in rare complications or long-term oncological outcomes. Therefore, our claims regarding oncological safety are limited to the pathological outcomes available; we cannot comment on recurrence or cancer-specific survival, which require longer follow-up. Fifth, subjective VAS scores were obtained from a single surgeon and may be influenced by expectation bias, despite being recorded immediately postoperatively. Additionally, our study lacks objective technical performance assessments, such as motion analysis or validated global rating scales (e.g., GEARS). Future studies incorporating such metrics, as advocated in recent consensus work on objective performance metrics in urologic surgery (24), would provide more robust evidence. Moreover, cost-effectiveness analyses comparing 2D, 3D, and robotic platforms in real-world practice would help guide resource allocation.

## 5 Conclusion

The transition from 2D to 3D laparoscopic radical cystectomy follows a structured, quantifiable learning curve. An experienced 2D surgeon requires approximately 30 cases to achieve visuomotor recalibration, during which operative performance remains comparable to prior 2D laparoscopic levels. Beyond this adaptation phase, significant improvements in pelvic-phase operative efficiency (≈20% reduction) and intraoperative safety (reduced blood loss) are realized, while pathological quality- reflected by stable lymph node yield and low positive margin rates- appears to be maintained. These findings provide a practical, evidence-based framework that may guide training programs and resource planning during the adoption of 3D laparoscopy in complex pelvic cancer surgery.

## Data Availability

The raw data supporting the conclusions of this article will be made available by the authors, without undue reservation.
